# The Relation of Coffee Consumption to Serum Uric Acid in Japanese Men and Women Aged 49–76 Years

**DOI:** 10.1155/2010/930757

**Published:** 2010-07-27

**Authors:** Ngoc Minh Pham, Daigo Yoshida, Makiko Morita, Guang Yin, Kengo Toyomura, Keizo Ohnaka, Ryoichi Takayanagi, Suminori Kono

**Affiliations:** ^1^Department of Preventive Medicine, Graduate School of Medical Sciences, Kyushu University, Fukuoka 812-8582, Japan; ^2^Department of Geriatric Medicine, Graduate School of Medical Sciences, Kyushu University, Fukuoka 812-8582, Japan; ^3^Department of Medicine and Bio-Regulatory Science, Graduate School of Medical Sciences, Kyushu University, Fukuoka 812-8582, Japan

## Abstract

*Objective*. Few studies have suggested an inverse relation between coffee intake and serum concentrations of uric acid (UA), but none has addressed the relation in men and women separately. We examined the relation between coffee intake and serum UA levels in free-living middle-aged and elderly men and women in Fukuoka, Japan. *Methods*. Study subjects were derived from the baseline survey of a cohort study on lifestyle-related diseases, and included 11.662 men and women aged 49–76 years; excluded were those with medication for gout and hyperuricemia, use of diuretic drugs, and medical care for cancer or chronic kidney disease. Statistical adjustment was made for body mass index, alcohol use, hypertension, diabetes mellitus, and other factors. *Results*. There were inverse associations of coffee consumption with serum UA concentrations and hyperuricemia in men regardless of adjustment for covariates. Women showed a statistically significant, but weaker, inverse association between coffee and serum UA levels after allowance for the confounding factors. *Conclusion*. The findings add to evidence for a protective association between coffee intake and hyperuricemia.

## 1. Introduction

Uric acid (UA) is the end product of purine degradation and serum UA concentrations are determined by the production and excretion of urate. Hyperuricemia is a predictor for gout [1] and has been related to increased risks of cardiovascular disease, stroke, and metabolic syndrome [2–4]. Of several behavioral and clinical correlates of serum UA, serum creatinine, obesity, and high alcohol consumption have most consistently been related to elevated concentrations of serum UA [5–7]. Serum UA concentrations are also increased in individuals who frequently consume a high amount of sugar-sweetened soft drinks [8]. A limited number of studies reported that coffee consumption was associated with lower concentrations of UA [9, 10] and a decreased risk of gout [11]. Coffee is one of the beverages commonly consumed in Western society [12] and is consumed daily by nearly half of the adults in Japan [13]. We examined the relation of coffee consumption to serum UA in a large population of free-living men and women in Japan.

## 2. Materials and Methods

### 2.1. Study Subjects

The subjects were men and women aged between 49–76 years who participated in the baseline survey of a cohort study on lifestyle-related diseases. Details of the baseline survey have been described elsewhere [14]. The study was approved by the Ethics Committee of the Faculty of Medical Sciences, Kyushu University. Written informed consent was obtained from all subjects. In brief, residents aged 50–74 years in the East Ward of Fukuoka City were recruited from February 2004 to August 2007. A total of 12,957 subjects participated in the survey, and 9 persons withdrew the informed consent afterwards. As of 1 February 2010,12.948subjects (5.817 men and 7.131 women) were study participants, with an estimated participation rate of 24%.Three women aged 49 years were included because of a mistake in recording the date of birth, and the inclusion of those aged 75 or 76 years was due to an interval in time from reference to the resident registry to participation in the survey.

Excluded in the present study were those with medication for gout or hyperuricemia (*n* = 524), use of diuretic drugs (*n* = 298), serum UA >12 mg/dL (*n* = 15), and medical care for cancer (*n* = 498) or chronic kidney disease (*n* = 56). Chronic kidney disease was defined as reported chronic renal failure or estimated glomerular filtration rate (eGFR) <30 mL/minute/1.73 m^2^ (*see below*). Some of these subjects had two or more conditions for exclusion. After further exclusion of subjects with missing information on covariates (*n* = 16), 11.662 subjects (4.964 men and 6.698 women) remained in the present analysis.

### 2.2. Laboratory Measurements

Recorded information was referred to regarding serum biochemical measurements including UA and creatinine (Cr) if the measurements had been done in the past year. When recorded information was not available, 5 mL of venous blood was taken for the measurements, and serum samples frozen in dry ice were shipped to an external laboratory (SRL, Hachiohji, Japan). Serum concentrations of UA were measured by the uricase-peroxidase method, and serum Cr levels were determined by the enzymatic creatinine assay method. Recorded measurements were available for 4.314 subjects (37%), and the remaining 7.348 (63%) donated venous blood for measurements at the baseline survey. Glycated hemoglobin (HbA_1c_) was determined for all the participants by the latex agglutination immunoassay at the above-mentioned external laboratory. GFR was estimated using the revised equation for Japanese population [15].

### 2.3. Assessment of Lifestyle Factors

A self-administered questionnaire was used to ascertain dietary and nondietary lifestyle factors, diseases under medical care, past history of selected diseases, use of drugs, and others. Food and beverage intake over the previous year was also assessed by a dietary questionnaire, which was a modified version of the food frequency questionnaire constructed by Tokudome et al. [16, 17]. Because only one item was used for alcohol consumption in the original questionnaire, the alcohol-related question was replaced with quantitative questions on 5 types of alcoholic beverages (sake, shochu, beer, whiskey, and wine). Furthermore, semiquantitative questions on consumptions of coffee and three types of tea were added. Consumptions of coffee and tea were elicited by closed-ended questions (almost null, 1-2, 3-4, or 5-6 cups per week, and 1–3, 4–6, 7–9, or ≥10 cups /day).

Caffeine intake was estimated from consumptions (cups/day) of coffee, green tea, black tea, and oolong tea, with the volume of one cup of each beverage assumed to be 150 mL. Caffeine contents per cup of these beverages (coffee 90 mg, black tea 45 mg, green tea 30 mg, and oolong tea 30 mg) were derived from the Food Composition Table in Japan [18]. Alcohol drinkers were defined as those who had consumed alcoholic beverages at least once per week over a period of one year or longer, and former alcohol drinkers were separated from lifelong abstainers. Ethanol intake was estimated for current drinkers according to the reported consumption frequencies and amounts of the five types of alcoholic beverages on average in the past year. Coffee, green tea, and alcohol intakes estimated by similar questions were shown to have a fairly good agreement with the intakes derived from a 28-day diet record [19]. Intake of seafoods was also estimated on the basis of consumption frequencies of 7 food items (fish, bone-edible small fish, canned tuna, shellfish, cuttlefish/octopus/shrimp/crab, fish eggs, and fish paste products) [16].

Smokers were defined as those who had ever smoked one or more cigarettes per day for at least one year or longer, with past smokers differentiated from life-long nonsmokers. Past and current smokers reported an average number of cigarettes smoked per day and total years of smoking. Questions on physical activity ascertained the amount of time for four types of work-related physical activities (standing, bicycling, walking, and strenuous labor), including commuting and domestic work, and for three types of leisure-time physical activities (light, moderate, and heavy) over the previous year. The intensity of each physical activity was determined in terms of metabolic equivalent (MET) value [20] and expressed as a sum of MET multiplied by time in hour spent in each activity.

### 2.4. Assessment of Clinical Factors

Height (cm) and weight (kg) were measured, and body mass index (BMI) was calculated by dividing weight by squared height (kg/m^2^). Systolic and diastolic blood pressure were measured by an automated digital device (HEM-707, OMRON, Kyoto) with subject in a sitting position at least for five minutes. Blood pressure readings were taken twice with an interval of at least one minute, and the second reading was used for the present study. Hypertension was defined if systolic blood pressure was ≥140 mmHg or diastolic blood pressure was ≥90 mmHg or if antihypertensive drugs were used. Diabetes mellitus status was defined if individuals reported current medication for diabetes mellitus or if HbA_1c_ was ≥6.5%.

### 2.5. Statistical Analysis

Statistical analyses were performed in men and women separately. We calculated age-standardized proportions of variables of interest according to categories of coffee intake by the direct method with age-specific (5-year class) numbers of men and women each as standard populations. Analysis of covariance was used to calculate adjusted mean concentrations of UA according to coffee or caffeine intake. Coffee intake was categorized into null consumption, <1, 1–3, 4–6, and ≥7 cups/day. Caffeine was divided into quintiles (<105, 105–194, 195–239, 240–309, and ≥310 mg/day). In multivariate models, we included the following potential confounding variables: age (continuous), BMI (continuous), smoking (never, past, and current smoking with a consumption of <20 or ≥20 cigarettes/day), alcohol drinking (never, past, and current drinking with a consumption of <30, 30–59, or ≥60 mL/day), work-related physical activity (quartiles of MET-hour/day), and leisure-time physical activity (quartiles of MET-hour/week), hypertension, diabetes mellitus, eGFR (<60, 60–89, or ≥90 mL/min/1.73 m^2^), and seafood intake (quartiles of daily consumption).

Odds ratios (OR) of hyperuricemia (≥7 mg/dL) in relation to coffee and caffeine intake were estimated by using logistic regression analysis. The definition of hyperuricemia was based on the standard cutoff point used in the United States [1]. The Wald method was used to estimate 95% confidence interval (CI). Trends in mean concentrations of serum UA and ORs of hyperuricemia across coffee or caffeine intake levels were evaluated by assigning ordinal values to categories of coffee or caffeine intake. A two-sided *P* value <  .05 was considered statistically significant. Statistical analyses were performed with SAS version 9.2 (SAS Institute, Cary, NC).

## 3. Results

Selected characteristics of the study subjects are summarized in Table 1. At least 1 cup of coffee per day was consumed by more than half of men and women (57%). Much larger proportions of men (78%) and women (87%) consumed green tea daily. Daily users of black tea and oolong tea were less frequent. Serum UA concentrations were greater in men than in women, as was the prevalence of hyperuricemia. Proportions of current cigarette smoking and alcohol use were greater in men than in women. Hypertension and diabetes mellitus were more frequent in men than in women. 

Distributions of potential confounding factors according to coffee consumption are shown in Table 2. In both men and women, those with higher intake of coffee were younger, and more likely to be smokers. Men with higher coffee intake were less likely to be alcohol drinkers while women showed an opposite association. BMI was greater in women, but not in men, with higher coffee consumption. Men with higher intake of coffee tended to consume less seafoods, whereas women showed no variation in seafood intake according to coffee consumption. There was no appreciable variation in the prevalence of diabetes mellitus and low eGFR according to coffee consumption in either men or women while hypertension was less frequent in men with high consumption of coffee. 

Age-adjusted and multivariate-adjusted means of serum UA were progressively lower with increasing consumption of coffee in men (Table 3). Age-adjusted mean of serum UA was 0.37 mg/dL lower in men consuming 7 cups or more of coffee per day than in those with a null consumption of coffee. The corresponding difference in the multivariate analysis was 0.27 mg/dL. In women, multivariate-adjusted mean showed a marginally significant inverse association with coffee consumption while no association was observed for age-adjusted mean concentration. Coffee intake also showed an inverse association with hyperuricemia in men, but not in women, regardless of adjustment for the covariates (Table 4). Men with intake of 4 or more cups of coffee per day had 30% lower prevalence odds of hyperuricemia compared with individuals with no consumption of coffee. In men, caffeine intake showed a weak inverse association with serum UA in the age-adjusted model (*P* for trend = .03), but not in the multivariate model (*P* for trend = .45). Caffeine intake was unrelated to serum UA in women and hyperuricemia in both sexes (data not shown).

## 4. Discussion

The present study showed evident inverse associations of coffee consumption with serum UA concentrations and hyperuricemia in men. Women also showed a statistically significant, but weaker, inverse association between coffee and serum UA levels. These inverse associations were independent of other factors for elevated serum UA and hyperuricemia, including BMI, smoking, alcohol use, hypertension, diabetes mellitus, and seafood intake.

Previously, lower concentrations of serum UA associated with coffee consumption were observed, with and without adjustment for confounding factors, in cross-sectional studies of men in Japan [9] and of both men and women combined in the United States [10]. Thus, the present findings corroborate the previous observation regarding coffee and serum UA [9, 10]. The present study first addressed the relation between coffee intake and serum concentrations of UA in women separately. The inverse association in women was not so evident as observed in men. A weaker association in women is probably ascribed to much lower concentrations of serum UA in women than in men. The difference in serum UA between men and women was 1.3 mg/dL while the age-adjusted difference in men between the highest category of coffee intake (≥7 cups/day) and null intake was no more than 0.4 mg/dL. An effect of coffee lowering serum UA, if any, would not be discernible in subjects who have inherently lower levels of serum UA. 

Caffeine is known to increase eGFR and renal blood flow, and it is possible that caffeine may enhance urinal excretion of UA. However, caffeine was found to be unrelated to serum concentrations of UA. The finding is consistent with an earlier observation [10] and lends further support to the hypothesis that compounds of coffee other than caffeine contribute to an inverse association between coffee and serum concentrations of UA [9, 10].

Increased insulin sensitivity has been proposed as a possible mechanism underlying an inverse association between coffee and serum UA [10]. There has been a strong, positive association between insulin resistance or hyperinsulinemia and hyperuricemia [21, 22] and insulin is shown to decrease eGFR levels [23, 24]. Intake of caffeinated and decaffeinated coffee was inversely related to plasma C-peptide concentrations [25]. It was recently shown that a major phenolic compound in coffee (chlorogenic acid) resulted in decreases in glucose and insulin concentrations after an oral glucose challenge [26]. Coffee may also contain substances which inhibit xanthine oxidase, an enzyme converting xanthine to uric acid, as speculated previously [9].

Advantages in the present study were a fairly large size of the study population, uniform measurements of lifestyle variables, and control for important confounders. Several weaknesses need to be discussed, however. A cross-sectional association does not necessarily indicate causality. Prevalent morbid conditions may affect UA concentrations and coffee consumption. For this reason, we excluded participants who had life-limiting morbid conditions such as cancer, severe chronic kidney disease and those under treatment for gout or hyperuricemia. A low participation (24%) in the survey was another concern in interpreting the findings. Selection bias may have been possible if the participation had been affected simultaneously by both coffee consumption and serum UA levels. As for serum UA concentrations, we used the measurements done at different laboratories among 37% of the study subjects. This may have resulted in an extraneous variation in the measurement, possibly diluting the true association. However, the measurements of routine blood biochemistry, including serum UA, have been standardized among clinical laboratories in Fukuoka Prefecture [27]. Decaffeinated coffee was not specifically distinguished from regular caffeinated coffee, but decaffeinated coffee is not common in Japan.

## 5. Conclusion

In a large cross-sectional study of free-living Japanese, coffee consumption was found to be related to lower concentrations of serum UA and a lower prevalence odds of hyperuricemia in men. Women also showed a weak association between coffee and serum UA concentrations. The findings add to evidence for a protective association between coffee intake and hyperuricemia.

## Conflicts of Interest

None declared.

## Figures and Tables

**Table 1:** Characteristics of the study subjects (*n* = 11.662).

Variable	Men (*n* = 4964)	Women (*n* = 6698)
Age (year), mean (SD)	62.6 (6.8)	62.1 (6.7)
Body mass index (kg/m^2^), mean (SD)	23.5 (2.8)	22.5 (3.1)
Current alcohol use, (%)	70.8	26.6
Alcohol (mL/d)*, median (IQR)	37 (18–57)	11 (6–20)
Current smoking, (%)	31.6	6.4
Cigarettes/d^†^, median (IQR)	20 (15–25)	15 (10–20)
Daily use of coffee, (%)	56.9	57.2
Daily use of green tea, (%)	78.1	87.3
Daily use of black tea, (%)	4.1	8.9
Daily use of oolong tea, (%)	12.9	14.0
Caffein intake (mg/d), median (IQR)	210 (109–270)	220 (136–284)
Seafood intake (g/d), median (IQR)	53 (36–74)	51 (38–68)
Work-related activity (MET-hr/d), median (IQR)	6 (2–14)	10 (6–18)
Leisure-time activity (MET-hr/w), median (IQR)	5 (2–15)	5 (1–14)
Hypertension, (%)	61.8	46.3
Diabetes mellitus, (%)	10.9	4.3
Serum creatinine (mg/dL), mean (SD)	0.8 (0.1)	0.6 (0.1)
eGFR (mL/minute/1.73 m^2^), mean (SD)	73.8 (14.0)	75.9 (14.7)
Serum uric acid (mg/dL), mean (SD)	5.9 (1.3)	4.6 (1.0)
Hyperuricemia, (%)	18.9	1.2

eGFR: estimated glomerular filtration rate; IQR: interquartile range; MET: metabolic equivalent; SD: standard deviation.

*Among current alcohol drinkers.

^†^Among current smokers.

**Table 2:** Age-adjusted means and proportions of possible confounders according to coffee consumption levels by sex*.

Variable	Coffee (cups/d)					*P* for trend^†^
	0	<1	1–3	4–6	≥7	
Men						
No. of subjects	846	1294	2216	508	100	
Age (year), mean	64.4	63.6	62.0	60.1	58.8	<.0001
Body mass index (kg/m^2^), mean	23.3	23.6	23.5	23.5	23.3	.88
Current alcohol use, %	73.2	73.1	70.3	63.9	55.6	<.0001
Current smoking, %	21.8	24.6	33.5	48.2	54.6	<.0001
High intake of seafood^‡^, %	27.1	26.6	25.3	21.7	18.6	.02
High job activity (≥17 MET-hr/d)^§^, %	22.4	19.8	20.6	18.8	21.3	.28
High leisure-time activity (≥15 MET-hr/w)^§^, %	25.5	26.3	25.8	27.6	20.5	.90
Hypertension, %	64.4	62.8	60.9	61.1	60.1	.04
Diabetes mellitus, %	11.8	9.9	10.3	15.9	14.3	.48
Low eGRF (<60 mL/min/1.73 m^2^), %	11.1	14.6	14.8	13.0	13.9	.10

Women						
No. of subjects	1054	1810	3270	496	68	
Age (year), mean	63.9	63.2	61.4	59.0	58.6	<.0001
Body mass index (kg/m^2^), mean	22.3	22.5	22.6	23.0	23.2	<.0001
Current alcohol use, %	19.2	24.8	29.2	28.0	22.4	<.0001
Current smoking, %	3.4	4.4	6.3	15.1	15.5	<.0001
High intake of seafood^‡^, %	25.8	26.2	23.8	22.7	25.1	.29
High job activity (≥17 MET-hr/d)^§^, %	26.3	25.7	26.5	28.6	18.9	.45
High leisure-time activity (≥15 MET-hr/w)^§^, %	23.2	22.8	25.2	25.0	15.7	.13
Hypertension, %	46.6	46.9	46.3	44.5	40.1	.33
Diabetes mellitus, %	4.5	4.6	3.8	4.6	1.0	.27
Low eGRF (<60 mL/min/1.73 m^2^), %	10.6	9.7	9.4	8.8	10.3	.21

eGFR: estimated glomerular filtration rate; MET: metabolic equivalent.

*Means were adjusted for age (as a continuous variable), and proportions were adjusted for 5-year age class using the direct method with total men and women each as standard populations. ^†^Analysis of variance and analysis of covariance were used for continuous variables; the Mantel-Haenszel method was applied for age-standardized proportions. ^‡^The highest one-fourth in men (≥74 g/d) and in women (≥68 g/d). ^§^The highest one-fourth in the distribution.

**Table 3:** Serum concentrations of uric acid according to coffee intake in men and women.

Coffee (cups/d)	No. of subjects	Age-adjusted mean (95% CI)	Multivariate-adjusted mean (95% CI)*
Men			
0	846	5.92 (5.84–6.01)	5.96 (5.88–6.04)
<1	1294	5.91 (5.84–5.98)	5.87 (5.81–5.94)
1–3	2216	5.85 (5.79–5.90)	5.83 (5.79–5.88)
4–6	508	5.72 (5.61–5.83)	5.79 (5.68–5.89)
≥7	100	5.55 (5.31–5.80)	5.69 (5.47–5.92)
Trend^†^		*P * = .0003	*P * = .0016

Women			
0	1054	4.55 (4.49–4.61)	4.58 (4.52–4.64)
<1	1810	4.61 (4.56–4.65)	4.61 (4.57–4.66)
1–3	3270	4.57 (4.54–4.61)	4.57 (4.54–4.60)
4–6	496	4.54 (4.45–4.63)	4.48 (4.39–4.56)
≥7	68	4.63 (4.40–4.87)	4.54 (4.32–4.76)
Trend^†^		*P * = .94	*P * = .05

CI: confidence interval.

*Adjusted for age (continuous), BMI (continuous), smoking (never, past, <20, or ≥20 cigarettes/d), alcohol use (never, past, <30, 30–59, or ≥60 mL/d), work-related physical activity and leisure-time physical activity (each categorized at quartiles), hypertension, diabetes, eGFR (<60, 60–89, or ≥90 mL/minute/1.73 m^2^), and seafood intake (quartiles of daily intake for each sex).

^†^ *P* values were derived from multiple linear regression analysis with ordinal values assigned to categories of coffee consumption.

**Table 4:** Age-adjusted and multivariate-adjusted odds ratios (95% CI) of hyperuricemia according to coffee consumption in men and women.

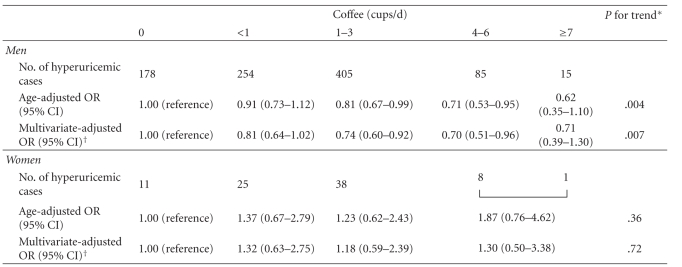

CI: confidence interval; OR: odds ratio.

*P values were obtained by assigning ordinal values to categories of coffee consumption.

^†^Adjusted for age (continuous), BMI (continuous), smoking (never, past, <20, or ≥20 cigarettes/d), alcohol use (never, past, <30, 30–59, or ≥60 mL/d), work-related physical activity and leisure-time physical activity (each categorized at quartiles), hypertension, diabetes, eGFR (<60, 60–89, or ≥90 mL/minute/1.73 m^2^), and seafood intake (quartiles of daily intake for each sex).

## Abbreviations

eGFR: Estimated glomerular filtration rate
MET: Metabolic equivalent
UA: Uric acid
SD: Standard deviation.
